# Polymorphisms of uridine glucuronosyltransferase gene and irinotecan toxicity: low dose does not protect from toxicity

**DOI:** 10.3332/ecancer.2014.428

**Published:** 2014-05-12

**Authors:** Marianna Tziotou, Vassiliki Kalotychou, Anna Ntokou, Revekka Tzanetea, Iakovos Armenis, Marianna Varsou, Konstantinos Konstantopoulos, Nicolas Tsavaris, Yannis Rombos

**Affiliations:** 1 1st Department of Internal Medicine, University of Athens, School of Medicine, Laikon General Hospital, 17 AgiouThoma str, 11527, Athens, Greece; 2 Department of Pathophysiology, Oncology Unit, University of Athens, School of Medicine, Laikon General Hospital, 17 AgiouThoma str, 11527, Athens, Greece

**Keywords:** dose, irinotecan, toxicity, uridine glucuronosyltransferase (UGT)

## Abstract

Uridine glucuronosyltransferase (*UGT*) gene polymorphisms have been linked to irinotecan toxicity. Our purpose was to study the association between UGT1A1*28, UGT1A7*2, and UGT1A7*3 polymorphisms and irinotecan toxicity in Greek patients receiving low-dose weekly irinotecan. Blood samples were collected for 46 patients. DNA was extracted and *UGT1A1* promoter and *UGT1A7* exon 1 genotyping was carried out. Laboratory tests and physical examination were performed on regular basis for the assessment of toxicity. UGT1A1*28 was significantly correlated with both haematologic and non-haematologic toxicity. Moreover, patients carrying *UGT1A7* polymorphisms had significant incidence of toxicity. To conclude, *UGT* polymorphisms play a role in the toxicity of irinotecan, even if the drug is administered in low doses. The genotyping test may be a useful tool for the management of patients who are going to receive irinotecan.

## Introduction

Pharmacogenomics studies the interaction between the genome and drugs on their way through the human body. To achieve their therapeutic role, drugs interact with multiple substances, such as enzymes. DNA polymorphisms may affect genes coding for enzymes in terms of quality or quantity of the enzyme produced. Thus, DNA polymorphisms may have an impact on the metabolism of drugs, especially chemotherapy. As the majority of chemotherapeutic agents have a narrow therapeutic range, even small defects in their metabolism may affect toxicity.

Irinotecan is an example of the above situation. Irinotecan is metabolised by carboxylesterases to form its active metabolite, SN-38. This is further glucuronated in the liver by glucuronosyltransferases and it forms the inactive metabolite SN-38G, which is eliminated mainly via the bile into the intestine. In the intestine, SN-38G is deconjucated back to SN-38 by bacterial β-glucuronidases. SN-38 is reabsorbed in the systemic circulation (enterohepatic circulation of SN-38). Glucuronosyltransferases expressed in the gut play a role in the reglucuronidation and detoxification of SN-38 in the intestine. The glucuronosyltransferase *UGT1A1*, which is primarily responsible for the glucuronidation of SN-38 in the liver, is also responsible for the conversion of indirect to direct bilirubin. Polymorphisms of the gene encoding *UGT1A1* may lead to reduced enzyme expression and reduced catalytic activity. UGT1A1*28 polymorphism is about a TA insertion in the TATA box of the promoter of the gene (seven instead of six TA repeats), (TA)7 instead of (TA)6 UGT1A1*1. The elongated TATA box in the promoter region results in reduced gene expression of about 30%. Approximately 10% of Caucasians are homozygous UGT1A1*28/UGT1A1*28 [(TA)_7_/(TA)_7_] for this polymorphism which leads to Gilbert’s syndrome. These patients cannot glucuronate indirect to direct bilirubin sufficiently and may present with mild indirect hyperbilirubinemia. These patients, for the same reason, have a reduced glucuronidation of SN-38, and thus a higher exposure to the active metabolite. This exposure has been linked to higher toxicity of irinotecan [1–4]. Polymorphisms of the gene encoding the extra hepatic glucuronosyltransferase *UGT1A7*, such as UGT1A7*2 and UGT1A7*3, may also lead to elevated toxicity due to reduced reglucuronidation of SN-38 in the intestine [5, 6]. Eighty-seven per cent of Caucasians carry at least one of the UGT1A7*2, UGT1A7*3, and UGT1A7*4 polymorphisms, the last being rather rare [7].

Since 2005, the US Food and Drug Administration (FDA) has recommended that a reduced initial dose of irinotecan should be considered for patients known to be homozygous for the UGT1A1*28 allele, because they are at increased risk for neutropenia following irinotecan treatment. However, genotyping for irinotecan toxicity has not been put into routine clinical practice. Furthermore, several studies failed to demonstrate any significant relationship between these *UGT* polymorphisms and irinotecan toxicity [8–11], and a meta-analysis concluded that the risk of toxicity was similar for patients with all genotypes at low doses of the drug [12]. In 2010, another meta-analysis concluded that UGT1A1*28/*28 genotype was associated with an increased risk of neutropenia in low doses as well. Moreover, it associated the homozygous and the heterozygous genotype with the risk of severe diarrhoea at medium and high doses [13, 14]. A recent meta-analysis showed that homozygous and heterozygous patients were at increased risk of neutropenia regardless of the dose of irinotecan administered. Homozygous patients were also at increased risk of diarrhoea when receiving medium or high doses, but not low doses, of the drug [15]. These results confirm the need for further studies, to determine the optimal dose for each patient and the need for genotyping.

Our purpose was to examine the association between irinotecan toxicity and UGT1A1*28, UGT1A7*2, and UGT1A7*3 polymorphisms in Greek patients with colorectal cancer receiving low-dose irinotecan treatment. These polymorphisms were selected for analysis as their prevalence is high among Caucasians and the results of previous studies were inconclusive. Information of this type, to the best of our knowledge, is lacking for Greek subjects.

## Patients and methods

Forty-six patients (23 male and 23 female) were studied from January 2009 to July 2012. Patients were eligible if they suffered from metastatic colorectal cancer treated with irinotecan–leucovorin–5-FU. The median age was 62.3 years (range: 33–78). Thirty-nine patients (84.8%) had performance status (Karnofsky) 70–90%, and seven (15.2%) 100%. The primary tumour site was the colon in 41 patients (89.1%), the rectum in four (8.7%), and unknown in one patient. The primary tumour was resected in 43 patients (93.5%) (Table 1).

All of them received, according to Gennatas *et al* [16], irinotecan 80 mg/m^2^ (30–90 min IV infusion), followed by leucovorin 200 mg/m^2^ bolus IV and 5-fluorouracil 450 mg/m^2^ bolus IV on days 1, 8, 15, 22, 29, 36, and every eight weeks. Treatment was discontinued in progression or intolerable toxicity. Before treatment, patients received atropine 0.5 mg SC to prevent cholinergic syndrome and ondansetron 8 mg IV to prevent vomiting. Ondansetron and loperamide were prescribed, incase of vomiting and diarrhoea. G-CSF was used for the treatment of neutropenia and as prophylaxis when it was considered necessary to maintain chemotherapy, according to the EORTC guidelines. Treatment was postponed until recovery in cases of grade ≥1 haematologic toxicity and diarrhoea as well as in cases of any other toxicity of grade ≥2 (except alopecia). In cases of grade 3–4 toxicity the irinotecan dose was reduced by 20% and in the recurrence of toxicity a second dose reduction by 20% was decided. Patients were evaluated with physical examination and laboratory tests before each treatment. Toxicity was graded according to the National Cancer Institute Common Terminology Criteria for Adverse Events v3.0. The research was carried out according to the principles set out in the Declaration of Helsinki 1964 and all subsequent revisions. The protocol of the study was approved by the ethics committee of the participating institution and all patients gave written informed consent.

Peripheral blood was collected and DNA was extracted using the QIAamp DNA Blood midi kit (Qiagen GmbH, Hilden, Germany). *UGT1A1* genotyping was performed by means of PCR using primers F1: 5′CTTGGTGTATCGATTGGTTTTTG3′ and R1: 5′ TTTGCTCCTGCCAGAGGTTCG3′. The amplicons were resolved on 12% polyacrylamide gel electrophoresis. Two different fragment sizes were revealed depending on the number of (TA) repeats. A 71-bp fragment contained the (TA)6 motif assigned as UGT1A1*1 and a 73-bp fragment contained the (TA)7 motif assigned as UGT1A1*28. *UGT1A7* genotyping was performed on exon 1 by means of PCR. A 415-bp fragment was amplified using primers F2: 5′TTTGCCGATGCTCGCTGGACG3′ and R2: 5′GCTATTTCTAAGACATTTTTGAAAAAATAGGG3′. Direct sequencing was performed on the resulting product that spans the informative polymorphic sites, namely G115S, N129K, R131K, W208R, and E139D. Genotypes were assigned as UGT1A7*1 (G115, N129, R131, and W208), UGT1A7*2 (K129 and K131), UGT1A7*3 (K129, K131, and R208), and UGT1A7*4 (R208).

Associations between genotypes of *UGT1A1*, *UGT1A7*, and baseline patients’ characteristics (age, sex, performance status, primary tumour site, previous chemotherapy, and metastatic sites) as well as with toxicities, dose delays, and dose reductions were investigated by chi-squared statistic with continuity correction. Ninety-five per cent confidence intervals for proportions indicating the magnitude of association are presented in case of statistically significant results. Evaluation of any toxicities, dose delays and dose reductions was based on the incidence of the event occurred upon the total weeks of treatment. One-way analysis of variance (ANOVA) was employed to investigate differences between the genotypes and laboratory parameters as transformed in logarithmic scale when needed. All tests were two-sided and the level of statistical significance was set at 5%.

## Results

*UGT1A1* genotyping revealed 22/46 patients (47.8%) as homozygotes UGT1A1*1/*1, 20/46 patients (43.5%) as heterozygotes UGT1A1*1/*28, and 4/46 patients (8.7%) as homozygotes for UGT1A1*28/*28. *UGT1A7* genotyping revealed the following genotypes with the equivalent frequencies, UGT1A7*1/*1 4/46 (8.7%), UGT1A7*1/*2 12/46 (26.1%), UGT1A7*1/*3 15/46 (32.6%), UGT1A7*1/*4 1/46 (2.2%), UGT1A7*2/*2 3/46 (6.5%), UGT1A7*2/*3 7/46 (15.2%), and UGT1A7*3/*3 4/46 (8.7%) (Table 2).

We considered serious toxicity, toxicity of grade ≥3 according to the National Cancer Institute Common Terminology Criteria for Adverse Events v3.0. Patients heterozygous for the UGT1A1*28 polymorphism, UGT1A1*1/*28, had significantly higher incidence of leucocytopenia (*p* = 0.0002), neutropenia (*p* = 0.0146), thrombocytopenia (*p* = 0.0032), febrile neutropenia (*p* = 0.0002), and overall haematologic toxicity (*p* < 0.00001), than those with UGT1A1*1/*1 and UGT1A1*28/*28 genotype. Homozygous UGT1A1*28/*28 patients experienced haematologic toxicity, especially leucocytopenia and neutropenia, but because of the few events analysed the results were not statistically significant. The incidence of anaemia did not correlate with genotype. As far as non-haematologic toxicity is concerned, patients UGT1A1*1/*28 and UGT1A1*28/*28 had significantly higher incidence of both diarrhoea and total non-haematologic toxicity than UGT1A1*1/*1 patients (*p* = 0.0002 and *p* = 0.02204, respectively) (Table 3).

Apart from UGT1A1*28, UGT1A7*2, and UGT1A7*3 were studied. One patient carried the rare allele UGT1A7*4. Regarding haematologic toxicity there was significant difference between genotypes who experienced, UGT1A7*1/*3, UGT1A7*2/*3, UGT1A7*3/*3, and those who did not experience, UGT1A7*1/*4 and UGT1A7*2/*2, toxicity. Moreover, genotype UGT1A7*1/*3 had a significantly higher incidence of toxicity than genotypes UGT1A7*1/*1 and UGT1A7*1/*2 (*p* = 0.0005). Concerning non-haematologic toxicity there was again significant difference between genotypes who experienced, UGT1A7*1/*2, UGT1A7*1/*3, UGT1A7*2/*3, UGT1A7*3/*3, and those who did not experience, UGT1A7*1/*1, UGT1A7*1/*4, UGT1A7*2/*2, toxicity (*p* = 0.00702) (Table 4).

Dose delays and dose reductions had no significant association with genotypes.

Genotype UGT1A1*28/*28 had significantly higher mean ( *p* = 0.043) and maximum ( *p* = 0.034) bilirubin rates during the entire course of therapy than UGT1A1*1/*28 and UGT1A1*1/*1, as it was expected. Data were transformed into logarithms and geometric means were 1.15 (95% confidence interval [0.17–7.54]) and 1.90 (95% confidence interval [0.21–17.50]), respectively.

Patients with haematologic toxicity only, had significantly lower baseline and mean haemoglobin (*p* = 0.003 and *p* = 0.023, respectively) as compared with all other cases.

## Discussion

The purpose of this study was to investigate whether *UGT1A1* and *UGT1A7* genotypes, more common in Caucasians, correlate with irinotecan toxicity, even at low doses of the drug (80 mg/m^2^).

Our results show that in our population the presence of UGT1A1*28 polymorphism was associated with both haematologic and non-haematologic toxicity. Heterozygous UGT1A1*1/*28 patients encountered significant haematologic and non-haematologic toxicity in accordance with the results of previous studies, which correlated UGT1A1*1/*28 genotype with toxicity [1, 2]. As far as it concerns homozygous UGT1A1*28/*28 patients, they experienced significant non-haematologic toxicity, especially diarrhoea. Moreover, UGT1A1*28/*28 patients experienced leucocytopenia and neutropenia, although this is not statistically significant, probably due to the small number of the events analysed, as we only had four homozygous patients.

The first studies published on this subject led to the FDA recommendation on the dose recommended to UGT1A1*28/*28 patients. However, conflicting results from subsequent studies are opposing such genotyping in clinical practice. Lankisch *et al* [5] did not correlate UGT1A1*28 polymorphism with toxicity in patients receiving low-dose irinotecan regimens (80 mg/m^2^/wk). Schulz *et al* [9] mentioned no significant effect of UGT1A1*28 polymorphism on irinotecan toxicity in patients treated with low-dose irinotecan (80 mg/m^2^/wk, modified FOLFIRI – modified IROX) as well. In a meta-analysis, Hoskins *et al* [12] concluded that the risk of toxicity was not statistically different between UGT1A1*28/*28 and UGT1A1*1/*1 at low irinotecan doses and patients receiving lower doses should not be genotyped. Deeken *et al* [17] suggested commencing therapy at higher doses for a better response and dose reduction if toxicity appeared. On the other hand, Toffoli *et al* [18] showed that the recommended dose of irinotecan in FOLFIRI (180 mg/m^2^) is lower than the one that can be tolerated by UGT1A1*1/*1 and UGT1A1*1/*28 patients. This poses the question whether higher doses confer a survival advantage to patients. However, Toffoli *et al* [18] did not study UGT1A1*28/*28 patients.

Our results for *UGT1A7* polymorphisms correlate them, and especially UGT1A7*3, with both haematologic and non-haematologic toxicity, as patients carrying the UGT1A7*3 allele seemed to have higher incidence of toxicity. Cecchin *et al* [6] in agreement to our results, concluded that UGT1A7*3/*3 genotype is the only predictive marker of severe haematologic toxicity after the first cycle of chemotherapy. UGT1A7*3 interference with non-haematologic toxicity is referred by Martinez-Balibrea *et al* [4] correlating UGT1A7*3/*3 genotype with higher risk of severe diarrhoea at the end of the first cycle of chemotherapy. We therefore propose that results for UGT1A1*28, UGT1A7*2, and UGT1A7*3 alleles, indicate that they may be used as useful markers for predicting haematologic and non-haematologic toxicity in irinotecan treated patients.

Apart from genetic factors altering the drug metabolism, it is possible that epigenetic changes in colon cancer cells play a role in the expression of *UGT*s and the metabolism of irinotecan by the tumour, influencing treatment toxicity and efficacy [19]. Moreover, DNA methylation at selected CpG sites in the promoter region of the *UGT1A1* gene, in liver, may influence the catalytic activity of the underlying enzyme [20].

We would also like to note that irinotecan is given in combination with 5-FU, which is a drug that may also cause haematologic and gastrointestinal toxicity. There is, therefore, a possibility that 5-FU may play a role in toxicity.

Dose delays and dose reductions did not show any association with genotypes despite the fact that they indirectly refer to toxicity. Other factors such as performance status, age, sex, primary tumour resection, previous chemotherapy, and metastatic sites were analysed and did not have any association with toxicity or genotype. Patients who suffered from haematologic toxicity had lower baseline and mean haemoglobin. None of the other basic laboratory tests had any association with toxicity. Homozygous UGT1A1*28/*28 patients had significantly higher mean and maximum bilirubin rates, but no other biochemical marker had any correlation with genotype.

We did not conduct linkage disequilibrium analysis as Kohle *et al* [21] has already showed frequent co-occurrence of UGT1A1*28 and UGT1A7*3 alleles. Nevertheless, we would like to note that three out of four UGT1A1*28/*28 patients in our sample were also UGT1A7*3/*3.

According to the results of the present study, we propose *UGT* genotyping, especially for UGT1A1*28, UGT1A7*2, and UGT1A7*3 alleles in all patients, regardless dose, as even low doses (80 mg/m^2^) of irinotecan may increase toxicity. Genotyping can help in personalising patient’s management thus reducing toxicity and subsequent financial costs (antibiotics and growth factors). Further studies are needed in optimising the dose for each individual.

## Conclusions

Despite previous studies [5, 9, 12], our results show that irinotecan, even in low doses, can cause serious toxicity in patients carrying *UGT* polymorphisms. However, some ten years after the first reports on the role of *UGT*s on irinotecan toxicity, many questions remain to be answered. New, larger studies are needed before utilising this knowledge in everyday practice.

## Figures and Tables

**Table 1.** Patients’ characteristics.

Characteristics	Number of patients	%
Sex		
Male	23	50
Female	23	50
Age		
Median	62.3	
Range	33–78	
Performance Status (Karnofsky)		
70–90%	39	84.8
100%	7	15.2
Primary site		
Colon	41	89.1
Rectum	4	8.7
Unknown	1	2.2
Primary tumour resected		
Yes	43	93.5
No	3	6.5

**Table 2.** Genotype frequencies.

Genotype	Number of patients	%
*UGT1A1*		
UGT1A1*1/*1	22	47.8
UGT1A1*1/*28	20	43.5
UGT1A1*28/*28	4	8.7
*UGT1A7*		
UGT1A7*1/*1	4	8.7
UGT1A7*1/*2	12	26.1
UGT1A7*1/*3	15	32.6
UGT1A7*1/*4	1	2.2
UGT1A7*2/*2	3	6.5
UGT1A7*2/*3	7	15.2
UGT1A7*3/*3	4	8.7

**Table 3.** Correlation of UGT1A1*28 with toxicity (haematologic and non-haematologic).

Haematologic toxicity (Feb.Neut./Hb/WBC/Neu/Plts)*						
		Gr 0–2 events^∞^	Gr 3–4 events^╒^	95% CI Gr 3–4^╕^	Wks ther ^┼^	*p*-value
**Genotype**	*1/*1	579 (98.3%)	10 (1.7%)	0.93–3.09%	589	<0.00001
	*1/*28	347 (87.4%)	50 (12.6%)	9.69–16.22%	397	*X*^2^ = 55.024,
	*28/*28	97 (98.0%)	2 (2.0%)	0.62–7.04%	99	df = 2
**Overall**		1023 (94.3%)	62 (5.7%)		1085	

Non-haematologic toxicity (diarrhoea/vomiting/nausea)						
		Gr 0–2 events^∞^	Gr 3–4 events^╒^	95% CI Gr 3–4^╕^	Wks ther ^┼^	p-value
**Genotype**	*1/*1	564 (95.8%)	25 (4.2%)	2.90–6.20%	589	0.02204
	*1/*28	364 (91.7%)	33 (8.3%)	5.99–11.45%	397	*X*^2^ = 7.63,
	*28/*28	91 (91.9%)	8 (8.1%)	4.20–15.16%	99	df = 2
**Overall**		1019 (94.9%)	66 (5.1%)		1085	

* Haematologic toxicity (febrile neutropenia/haemoglobin/white blood cells/platelets).

∞ Grade 0–2 events.

╒ Grade 3–4 events.

╕ 95% confidence interval grade 3–4.

┼ Weeks of therapy.

**Table 4.** Correlation of UGT1A7*2 and UGT1A7*3 with toxicity (haematologic and non-haematologic).

Haematologic toxicity (Feb.Neut./Hb/WBC/Neu/Plts)*						
		Gr 0–2 events^∞^	Gr 3–4 events^╒^	95% CI Gr 3–4^╕^	Wks ther ^┼^	*p*-value
**Genotype**	*1/*1	109 (98.2%)	2 (1.8%)	0.56–6.30%	111	0.0005
	*1/*2	290 (96.0%)	12 (4.0%)	2.30–6.82%	302	*X*^2^ = 24.083,
	*1/*3	294 (90.5%)	31 (9.5%)	6.81–13.23%	325	df = 6
	*1/*4	32 (100.0%)	0		32	
	*2/*2	60 (100.0%)	0		60	
	*2/*3	155 (91.2%)	15 (8.8%)	5.44–14.06%	170	
	*3/*3	83 (97.6%)	2 (2.4%)	0.73–8.15%	85	
**Overall**		1023	62		1085	

Non-haematologic toxicity (diarrhoea/vomiting/nausea)						
		Gr 0–2 events^∞^	Gr 3–4 events^╒^	95% CI Gr3–4^╕^	Wks ther^┼^	*p*-value
**Genotype**	*1/*1	111 (100.0%)	0 (0.0%)		111	0.00702
	*1/*2	281 (93.0%)	21 (7.0%)	4.61–10.40%	302	*X*^2^ = 17.701,
	*1/*3	303 (93.2%)	22 (6.8%)	4.53–10.04%	325	df = 6
	*1/*4	32 (100.0%)	0 (0.0%)		32	
	*2/*2	60 (100.0%)	0 (0.0%)		60	
	*2/*3	155 (91.2%)	15 (8.8%)	5.44–14.06%	170	
	*3/*3	77 (0.6%)	8 (9.4%)	4.90–17.51%	85	
**Overall**		1019	66		1085	

* Haematologic toxicity (febrile neutropenia/haemoglobin/white blood cells/platelets).

∞ Grade 0–2 events.

╒ Grade 3–4 events.

╕ 95% confidence interval grade 3–4.

┼ Weeks of therapy.
